# Prospective real-world experience with risankizumab in difficult-to-treat Crohn’s disease: results from the Dutch Initiative on Crohn and Colitis Registry

**DOI:** 10.1093/ibd/izag045

**Published:** 2026-04-04

**Authors:** Loriane M M Verleye, Marieke J Pierik, Annemarie C de Vries, Fiona D M van Schaik, Andrea E van der Meulen-de Jong, Willemijn A van Dop, Mark Löwenberg, Marijn C Visschedijk, Myrthe R Naber, Dianne G Bouwknegt, Tessa E H Römkens, Alexander G L Bodelier, Philip W Voorneveld, Bas Oldenburg, Lauranne A A P Derikx, Marjolijn Duijvestein, Zlatan Mujagić, the Dutch Initiative on Crohn and Colitis (ICC), Ingrid Willemen-Boemaars, Safioen Nabibaks, Rachel L West, Sandra Cosijns, Loes H C Nissen, Bindia Jharap, Miranda Bouwens, Steven Jeuring, Ilse Sour, Kyra Hermans, Malena Schlotter, Martine van Workum, Marthe Verwey, Anne Meyboom, Jael Smid, Herma H Fidder, Nynke A Boontje, Vince Biemans, Tessa Straatmijer

**Affiliations:** Department of Gastroenterology and Hepatology, Maastricht UMC+, 6229 HX Maastricht, the Netherlands; NUTRIM Institute for Nutrition and Translational Research in Metabolism, Maastricht University, 6229 ER Maastricht, the Netherlands; Department of Gastroenterology and Hepatology, Maastricht UMC+, 6229 HX Maastricht, the Netherlands; NUTRIM Institute for Nutrition and Translational Research in Metabolism, Maastricht University, 6229 ER Maastricht, the Netherlands; Department of Gastroenterology and Hepatology, Erasmus University Medical Center, 3015 GD Rotterdam, the Netherlands; Department of Gastroenterology and Hepatology, University Medical Center Utrecht, 3584 CX Utrecht, the Netherlands; Department of Gastroenterology and Hepatology, Leiden University Medical Center, 2333 ZA Leiden, the Netherlands; Department of Gastroenterology and Hepatology, Radboudumc, 6525 GA Nijmegen, the Netherlands; Department of Gastroenterology and Hepatology, Amsterdam UMC, 1100 DD Amsterdam, the Netherlands; Department of Gastroenterology and Hepatology, University Medical Center Groningen, 9713 GZ Groningen, the Netherlands; Department of Gastroenterology and Hepatology, University Medical Center Utrecht, 3584 CX Utrecht, the Netherlands; Department of Gastroenterology and Hepatology, University Medical Center Groningen, 9713 GZ Groningen, the Netherlands; Department of Gastroenterology and Hepatology, Jeroen Bosch Hospital, 5223 GW 's-Hertogenbosch, the Netherlands; Department of Gastroenterology and Hepatology, Amphia Hospital, 4818 CK Breda, the Netherlands; Department of Gastroenterology and Hepatology, Leiden University Medical Center, 2333 ZA Leiden, the Netherlands; Department of Gastroenterology and Hepatology, University Medical Center Utrecht, 3584 CX Utrecht, the Netherlands; Department of Gastroenterology and Hepatology, Erasmus University Medical Center, 3015 GD Rotterdam, the Netherlands; Department of Gastroenterology and Hepatology, Radboudumc, 6525 GA Nijmegen, the Netherlands; Department of Gastroenterology and Hepatology, Maastricht UMC+, 6229 HX Maastricht, the Netherlands; NUTRIM Institute for Nutrition and Translational Research in Metabolism, Maastricht University, 6229 ER Maastricht, the Netherlands

**Keywords:** pharmacoepidemiology, difficult-to-treat disease, real-world population

## Abstract

**Background:**

Risankizumab, a monoclonal antibody targeting interleukin-23, is approved for the treatment of Crohn’s disease (CD). Real-world evidence on its effectiveness remains limited. We assessed 1-year effectiveness and safety of risankizumab in a prospective cohort of patients with CD, emphasizing difficult-to-treat disease.

**Methods:**

Data were retrieved from the Dutch Initiative on Crohn and Colitis Registry. At baseline and at weeks 12, 24, and 52, clinical remission (Harvey-Bradshaw Index ≤4), biochemical remission (fecal calprotectin ≤250 µg/g and C-reactive protein levels ≤5 mg/L), systemic corticosteroids use, and safety outcomes were determined. The primary outcome was corticosteroid-free clinical remission (CSFCR) at week 24. Difficult-to-treat disease was defined as the failure of advanced therapies with at least 2 different mechanisms of action.

**Results:**

In total, 151 patients initiated risankizumab before August 1, 2025; 136 (90.1%) of 151 patients had difficult-to-treat disease. At baseline, 90 (59.6%) patients had Harvey-Bradshaw Index  >4. Of those, at week 24, 31 (50%) of 62 patients were in CSFCR; this rate was 53.3% when including all patients. Analysis including only patients with difficult-to-treat disease revealed that 51.8% of these patients were in CSFCR at week 24. Twenty-two (14.6%) patients discontinued treatment after a median of 183.5 days (Q1-Q3: 90.0-295.2 days), most following nonresponse. Regarding adverse events, 35.3 possibly and 16.6 probably risankizumab-related events per 100 person-years were reported.

**Conclusion:**

In this prospective multicenter study, risankizumab demonstrated favorable effectiveness and safety profile in patients with CD, including those with difficult-to-treat disease, bowel surgery, and stoma. These findings support the use of risankizumab in patients with difficult-to-treat disease.

## Graphical Abstract

**Graphical Abstract:**
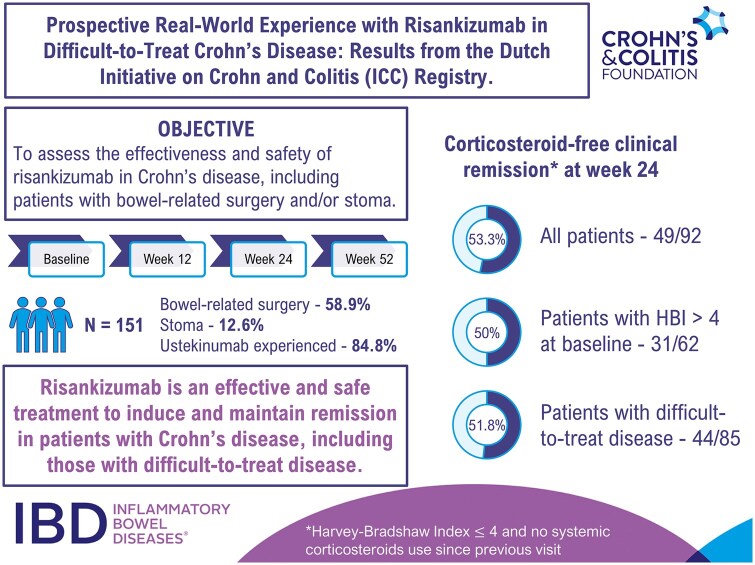

Key Messages *What is already known?*

Risankizumab is now approved for Crohn’s disease (CD), but real-world data remain limited. We aimed to complement and extend existing trial data using a nationwide prospective study.

*What is new here?*

We found that risankizumab is effective for CD, even in patients difficult-to-treat disease and patients with previous bowel-related surgery and stoma. Patients who achieved remission early tended to stay in remission until week 52. No new adverse reactions were observed.

*How can this study help patient care?*

Risankizumab benefits patients with CD, regardless of prior treatment failure. Healthcare providers should also consider risankizumab as a treatment option for patients with complex disease phenotypes or with previous failure of several treatments.

## Introduction

Risankizumab, a monoclonal antibody inhibiting the interleukin-23 p19 subunit, is one of the most recent pharmacotherapies available to induce and maintain remission in patients with Crohn’s disease (CD), an inflammatory bowel disease (IBD). It has demonstrated long-term efficacy and safety in other immune-mediated diseases, such as plaque psoriasis.^1^^,^^2^ In a meta-analysis of randomised controlled trials (RCTs) in patients with CD, Huang et al^3^ reported that risankizumab was efficacious and well tolerated. However, RCT results only partly reflect real-world clinical practice, as strict inclusion and exclusion criteria limit generalisability to the broader IBD population.^4^ In real-world settings novel pharmacological treatments in CD, such as risankizumab, are predominantly prescribed to patients who failed multiple previously available treatments. Among others, this population, characterized by difficult difficult-to-treat disease, is poorly represented in RCTs, and therefore the available data on effectiveness of risankizumab in these patients are scarce.

To date, only a few real-world studies have described the use of risankizumab in patients with CD, with the longest follow-up duration being 1 year.^5–8^ Although results were positive, these studies were retrospective in nature, which increases the risk of selection bias and inconsistent data quality; therefore, the strength of their conclusions is limited. To our knowledge, only 1 prospective study has been conducted: Zinger et al^9^ reported that more than half of the 134 included patients with active luminal disease achieved clinical remission by week 52. However, this was a monocenter study, and additional multicenter prospective cohorts are needed to validate and extend these findings.

Using the Dutch Initiative on Crohn and Colitis (ICC) Registry, a prospective, multicenter, nationwide observational registry for IBD-therapies, we aimed to evaluate the (long-term) effectiveness and safety of risankizumab in routine clinical care with up to 1-year follow-up, with a specific emphasis on patients with difficult-to-treat disease. In addition, we explored potential differences in treatment response across subgroups, including history of bowel-related surgery, and ustekinumab treatment failure.

## Methods

### Study design

Analyses were performed using data from the ICC Registry, a Dutch multicenter prospective cohort evaluating IBD therapies in routine clinical practice.^10^ Data were collected using a standardized electronic case report form within the ICC Registry, employing well-defined structured variables and a validated data model. The ICC Registry is currently running in 19 hospitals across the Netherlands, and patients are prospectively followed up to 10 years after treatment initiation.

### Participants

All patients included in the present study had CD as confirmed with endoscopy, histology, and/or radiology, were 16 years of age or older and initiated risankizumab treatment for CD before August 1, 2025. Patients were considered as having difficult-to-treat IBD if they failed advanced therapies with at least 2 different mechanisms of action.^11^

### Variables

At baseline, patient characteristics (age, sex, smoking status, body mass index, comorbidities), CD-specific characteristics (ie, age at diagnosis or Montreal classification), history of bowel-related surgery (eg, ileocecal resection, partial resection), presence of a stoma, and prior use of advanced therapies (ie, anti-tumor necrosis factor α, Janus kinase inhibitors, interleukin inhibitors, calcineurin inhibitors, or vedolizumab) were extracted from the ICC Registry.

### Outcome parameters and definitions

The primary outcome was corticosteroid-free clinical remission (CSFCR) at week 24. CSFCR was defined as clinical remission (Harvey-Bradshaw Index [HBI] ≤4) without the use of systemic corticosteroids since the previous visit (eg, for week 24: no use of systemic corticosteroids between week 12 and week 24). Patients without clinical data available but using systemic corticosteroids since the previous visit were included in the CSFCR analysis (ie, they cannot be in CSFCR if they used systemic corticosteroids since the previous visit). Although there is currently no validated method to assess clinical disease activity in patients with a stoma, bowel frequency was assessed by asking the patient how many times more than normal the stoma bag had to be emptied. Secondary outcomes included clinical remission, and biochemical remission, defined as fecal calprotectin (FC) levels ≤250 µg/g and C-reactive protein (CRP) levels ≤5 mg/L. All outcomes were assessed at weeks 12, 24, and 52, at the discretion of the IBD specialist and in accordance with local standard care. To minimize missing data and in consideration of the observational design of the study, variables were included only if their measurements fell within a predefined time window surrounding the time point of interest (Table S1, Figure S1).

Adverse events were labeled as possibly or probably related by the treating physician. Adverse events leading to therapy withdrawal were labeled as adverse events leading to treatment discontinuation. Infections that did not lead to antibiotic or antiviral medication were classified as mild infections. Infections requiring oral antibiotics of antiviral medication were classified as moderate infections, and infections leading to hospitalization and/or requiring intravenous administration of antibiotics or antiviral medication were classified as severe infections. Reasons for treatment discontinuation were systematically documented by the treating physician.

### Subgroup analyses

Analyses were performed in all patients included in the study. CSFCR was also assessed in a subgroup including only patients with HBI >4 at baseline, to avoid reverse causality. Comparison analyses were performed in patients with prior bowel-related surgery, number of prior advanced therapies, and prior use of ustekinumab.

### Statistical analysis

Normally distributed and non-normally distributed continuous variables were presented as mean ± SD or as median (Q1-Q3), respectively. Categorical variables were presented as absolute value and percentage. Treatment persistence was evaluated with the Kaplan-Meier estimator. Remission rates were calculated as the proportion of patients in specific remission relative to the total number of patients with available data. Patients who discontinued risankizumab due to primary or secondary nonresponse, adverse events, or personal request without achieving remission were classified as treatment failures and designated nonresponders at subsequent time points. Discontinuations associated with pregnancy were treated as censored cases at subsequent time points. Patients with insufficient follow-up at a given time point, irrespective of response status, were likewise considered censored cases and excluded from the analysis for that visit. Predictors of CSFCR at week 24 were assessed using binary logistic regression. Variables in the univariable analysis with *P* value <.2 were analyzed in a multivariable logistic regression model. Missing values were not imputed. Data were analyzed using RStudio version 2025.5.0.496. A 2-sided *P* value of .05 or lower was considered statistically significant.

### Ethics

The ICC Registry was approved by the Committee on Research Involving Human Subjects at the Radboudumc (4076). All participants provided written informed consent.

## Results

### Baseline characteristics

As August 1, 2025, 151 patients included in the ICC Registry initiated risankizumab treatment for CD. The majority were female (n = 94 [62.2%]); median disease duration at treatment initiation was 15.0 years (Q1-Q3: 8.0-22.0 years). Eighty-nine (58.9%) patients previously underwent bowel-related surgery and 19 (12.6%) had a stoma at treatment initiation. The median number of previous advanced therapies was 3 (3-4); 124 (84.8%) patients were ustekinumab experienced, and 71 (47.0%) patients used at least 4 different advanced therapies before initiating risankizumab (Table 1). Mean risankizumab treatment and follow-up duration were 349 ± 193 days and 396 ± 191 days, respectively.

**Table 1:** Baseline characteristics (n = 151).

**Female**	94 (62.2)
**Age at inclusion, y**	41.0 (31.5-55.0)
**Disease duration, y**	15.0 (8.0-22.0)
**Disease location at inclusion**	
**L1 (ileal)**	56 (37.1)
**L2 (colonic)**	28 (18.5)
**L3 (ileocolonic)**	61 (40.4)
**Unknown**	6 (4.0)
**L4 (upper GI modifier)**	3 (2.0)
**Disease behavior at inclusion**	
**B1 (nonstricturing nonpenetrating)**	95 (62.9)
**B2 (structuring)**	27 (27.9)
**B3 (penetrating)**	18 (11.9)
**Unknown**	11 (7.3)
**P (perianal disease modifier)**	22 (14.6)
**Smoking status**	
**Never smoked**	61 (40.4)
**Ex-smoker**	44 (29.1)
**Current smoker**	24 (15.9)
**Unknown**	22 (14.6)
**Previous advanced therapies**	
**None**	1 (0.7)
**≥1**	150 (99.3)
**≥2**	138 (91.4)
**≥3**	119 (78.8)
**≥4**	71 (47.0)
**Difficult-to-treat disease^a^**	136 (90.1)
**Ustekinumab experienced**	124 (84.8)
**Reason for ustekinumab discontinuation**	
**No response**	46 (35.9)
**Loss of response**	64 (50)
**Adverse events**	12 (9.4)
**Unknown**	6 (4.7)
**History of bowel resection**	89 (58.9)
**Stoma at inclusion**	19 (12.6)
**HBI**	6 (4, 9)
**Clinical remission^b^**	46 (30.5)
**Clinically active disease**	90 (59.6)
**Unknown**	15 (9.9)
**Biochemical assessment**	
**Biochemical remission^c^**	32 (23.7)
**Biochemically active disease**	100 (74.1)
**Unknown**	3 (2.2)
**FC, µg/g**	582.5 (230.8-1512.2)
**≤ 250 µg/g**	27 (17.9)
**> 250 µg/g**	77 (51.0)
**Unknown**	47 (31.1)
**CRP, mg/L**	5.0 (1.5-13.2)
**≤5 mg/L**	71 (47.0)
**>5 mg/L**	70 (46.4)
**Unknown**	10 (6.6)
**Concomitant medication**	
**Systemic corticosteroids**	32 (21.2)
**Advanced therapies^d^**	8 (5.3)
**Treatment initiation date**	
**Before February 1, 2025**	125 (82.8)
**Before August 1, 2024**	89 (58.9)

Values are n (%) or median (Q1-Q3).

Abbreviations: CRP, C-reactive protein; FC, fecal calprotectin; GI, gastrointestinal; HBI, Harvey-Bradshaw Index; IBD, inflammatory bowel disease.

a Prior failure of advanced therapies with at least 2 different mechanisms of action.

b HBI ≤4.

c FC levels ≤250 µg/g and/or CRP levels ≤5 mg/L, when available.

d Biologicals and small molecules.

### Clinical and biochemical endpoints

**Figure 1:**
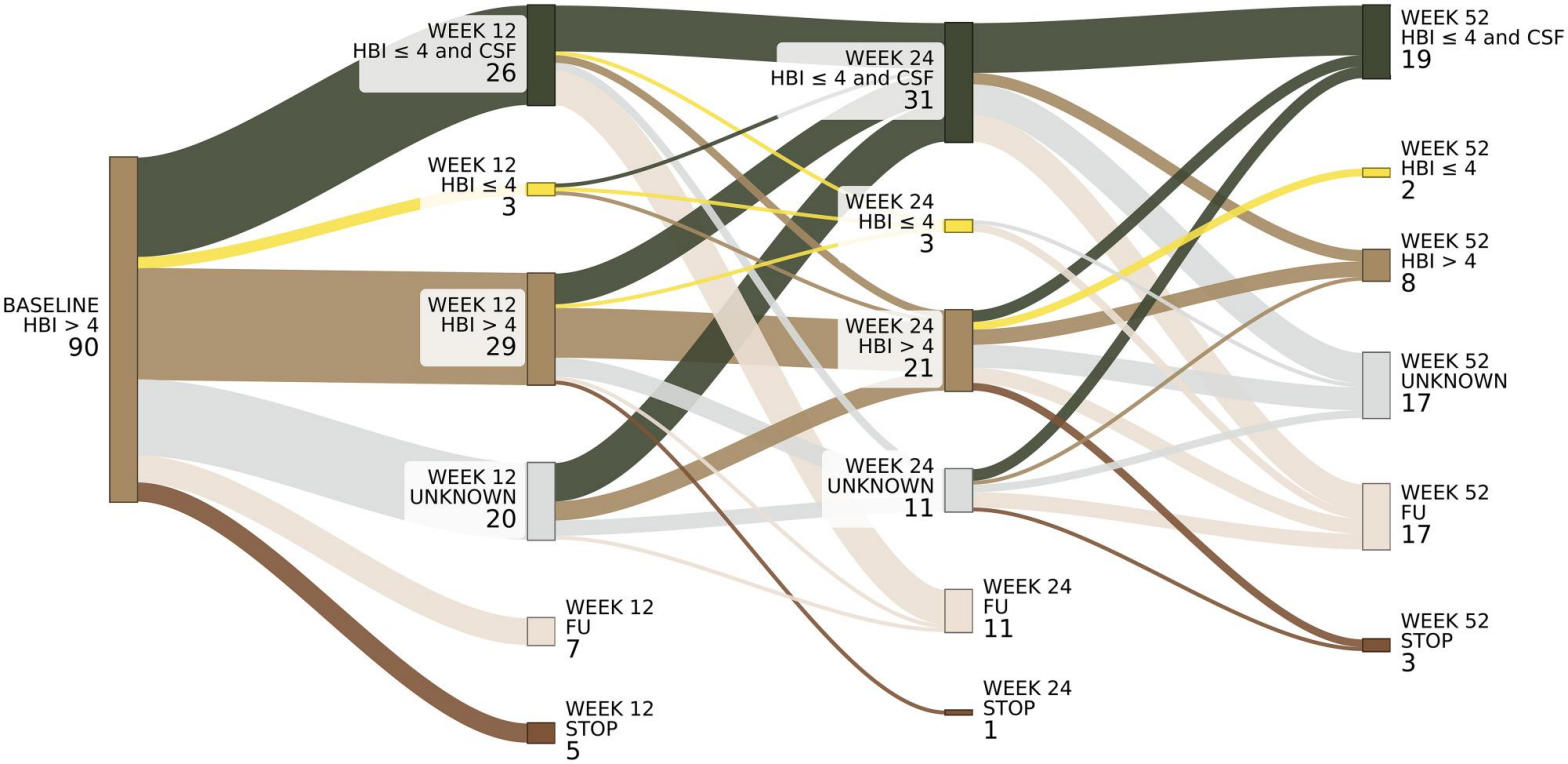
Flow of disease status at week 12, week 24 and week 52 in patients with clinically active disease (Harvey-Bradshaw Index [HBI] > 4) at treatment initiation. Patients in clinical remission but using systemic corticosteroids at the time point of interest were included in the clinical remission group, patients missing clinical data (UNKNOWN) at the specified time point were censored, patients with insufficient follow-up (FU) were censored at further time points, and patients who discontinued risankizumab (STOP) were not qualified as nonresponders in the current figure. CSF, corticosteroid-free.

**Figure 2:**
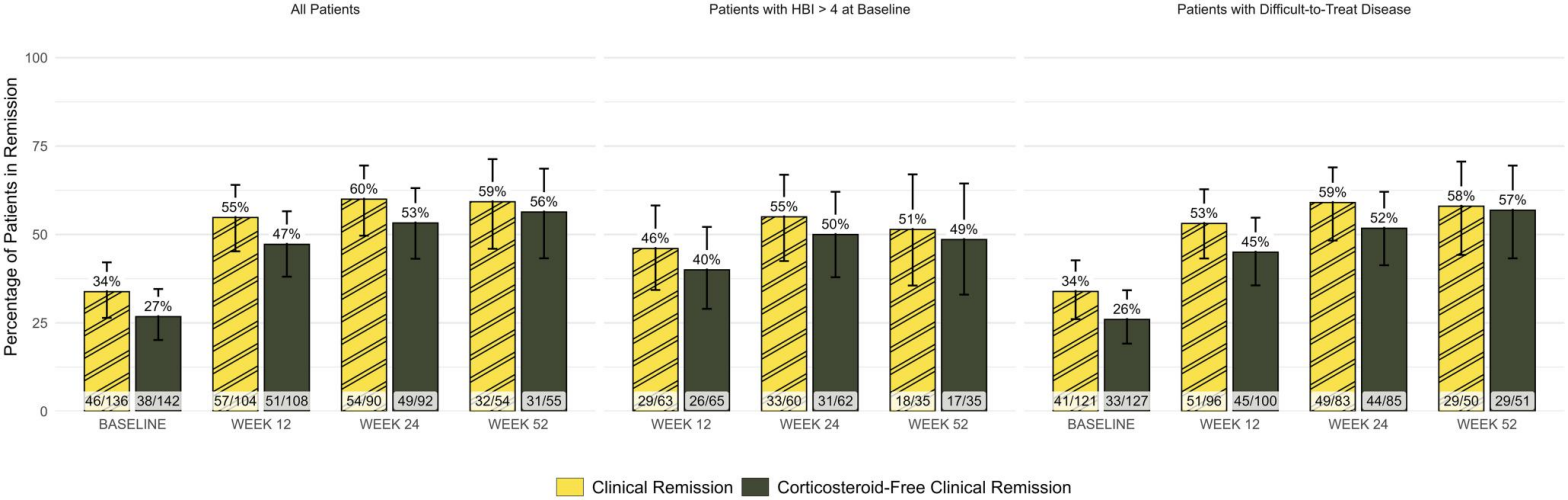
Percentage of patients in clinical remission and corticosteroid-free clinical remission at weeks 12, 24, and 52, in all patients, patients with clinical activity at baseline, and patients with difficult-to-treat disease. Vertical lines represent 95% confidence intervals, and patients missing clinical data at the specified time point but using systemic corticosteroids since the previous visit were included in the corticosteroid-free clinical remission analysis as nonresponder and censored in the clinical remission analysis. HBI, Harvey-Bradshaw Index.

HBI was available for 136 (90.1%) of 151 patients at baseline; 90 (66.2%) of these patients had clinically active disease and were included in the clinically active subgroup analysis. Results from the clinically active subgroup analysis showed that 26 (40%) of 65 patients were in CSFCR at week 12. At week 24, remission rates increased; 33 (55%) of 60 patients were in clinical remission, with 31 (50%) of 62 patients in CSFCR (Figures 1 and 2; Figure S2). When looking at the whole population, independent of clinical disease activity at baseline, results showed that 51 (47.2%) of 108 patients and 49 (53.3%) of 92 patients were in CSFCR at weeks 12 and 24, respectively (Figure 2; Figure S2).

At baseline, FC levels were available for 104 patients; 77 (74.0%) patients had FC levels >250 µg/g, and median FC levels were 582.5 µg/g (Q1-Q3: 230.8-1512.2 µg/g). CRP levels were available for 141 patients; 70 (49.6%) patients had CRP levels >5 mg/L, and median CRP levels were 5.0 mg/L (Q1-Q3: 1.5-13.2 mg/L). Altogether, 112 (75.7%) of 132 patients had biochemically active disease at treatment initiation. At week 24, 22 (31.9%) of 69 patients with biochemically active disease at baseline were in biochemical remission; when including all patients, 34 (39.1%) of 87 patients were in biochemical remission. When considering FC only, 12 (30.0%) of 40 patients with FC levels >250 µg/g at baseline were in biochemical remission at week 24.

Data on clinical and biochemical remission were both available for 99 and 80 patients at weeks 12 and 24, respectively. At week 12, 28 (28.3%) of 99 patients were in both clinical and biochemical remission, while 43 (43.4%) patients were either in clinical or biochemical remission, and 28 (28.3%) patients were nonresponders. At week 24, 27 (33.8%) of 80 patients were in both clinical and biochemical remission; 27 (33.8%) patients were either in clinical or biochemical remission, and 26 (32.5%) patients were nonresponders.

When defining remission as a composite of clinical and biochemical remission, 84 (68.3%) of 123 patients were in remission at week 12, including 70 (56.9%) patients in corticosteroid-free (CSF) remission. At week 24, 61 (62.9%) of 97 patients were in remission, with 51 (52.6%) patients in CSF remission. Remission rates at week 52 were similar, with 37 (62.7%) of 59 patients in remission. Thirty-six (61.0%) patients were in CSF remission.

### Patients with difficult-to-treat disease

Of the 151 included individuals, 90.1% (n = 136) were considered as having difficult-to-treat disease based on the number of previous advanced therapies. Only 8 (5.9%) patients were ustekinumab-naive, and 71 (52.2%) patients previously used at least 4 different advanced therapies. Eighty-one (59.6%) patients had a history of bowel-related surgery, and 19 (14.0%) patients had a stoma a treatment initiation.

At week 12, 45 (45%) of 100 patients were in CSFCR, including 41 patients with active disease at baseline. At week 24, 44 (51.8%) of 85 patients were in CSFCR. When considering composite remission, 55 (61.8%) of 89 patients were in remission at week 24. At week 52, 29 (56.9%) of 51 patients were in CSFCR (Figure 2).

At treatment initiation, 92 (67.6%) of 136 patients with difficult-to-treat disease were using systemic corticosteroids (n = 31) and/or had clinically active disease (n = 80). When including only those patients in the effectiveness analysis, 27 (41.5%) of 65 patients were in CSFCR at week 12. CSFCR rates increased to 47.5% (n = 29 of 61) and 54.3% (n = 19 of 35) at weeks 24 and 52, respectively.

Twenty-patients with difficult-to-treat disease discontinued risankizumab treatment, after a median treatment duration of 198 days (Q1-Q3: 86-301 days). Reasons for discontinuation included primary nonresponse (n = 8 of 20 [40.0%]), secondary nonresponse (n = 6 of 20 [30.0%]), and occurrence of adverse events (n = 6 of 20 [30.0%]).

### CSFCR at week 24: Comparison analysis

Comparison analysis based on history of bowel-related surgery, prior ustekinumab use, and number of prior advanced therapies was performed for all patients with at least 24 weeks of follow-up and in a subgroup of patients with clinically active disease at baseline. Results indicated that patients without history of surgery had a numerically higher rate of being in CSFCR at week 24 (65.1%) compared with patients with history of surgery (44.9%), with a mean difference of 20.2% (95% confidence interval [CI], 0.29 to 40.1). This mean difference increased to 29.2% and became statistically significant (*P* = .02; 95% CI, 5.4 to 52.9) when including only patients with clinically active disease at baseline. Patients who never used ustekinumab had also a higher rate of being in CSFCR at week 24 (61.5%) than ustekinumab-experienced patients (53.1%), with a mean difference of 8.4% (95% CI, −20.3% to 37.0%). However, results should be carefully interpreted, as group sizes varied largely. Mean difference in CSFCR at week 24 was 4.0% (95% CI, −9.4% to 34.2%) between patients who failed <4 advanced therapies (56.2%) and those who failed 4 or more advanced therapies (52.2%). Results did not differ when including only patients with clinically active disease at baseline.

We also examined in all patients with at least 24 weeks of follow-up whether being in clinical remission or using systemic corticosteroids at treatment initiation influenced the occurrence of being in CSFCR at week 24. Patients in clinical remission at baseline had a higher mean rate of being in CSFCR at week 24 (68.0%) compared with patients with clinically active disease at baseline (51.6%), although the mean difference was not statistically significant (95% CI, −38.5% to 5.3%). Patients who did not use systemic corticosteroids at treatment initiation showed a higher mean rate of being in CSFCR at week 24 (59.1%) compared with patients using systemic corticosteroids at baseline (33.3%). The mean difference of 25.8% was statistically significant (*P* = .037; 95% CI, 26.4% to 49.0%). When including only patients with clinically active disease at baseline, the mean difference of CSFCR rate decreased to 18.4% (54.2% vs 35.7%) and became statistically nonsignificant (*P* = .22; 95% CI, −10.3% to 47.2%).

### Subgroup analyses

#### Patients with a stoma

**Figure 3:**
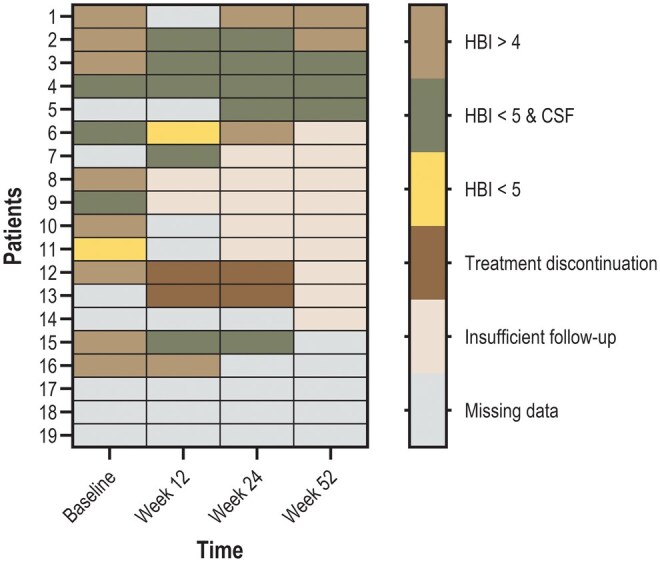
Lasagna plot depicting clinical disease activity status at baseline, week 12, week 24, and week 52 for patients with a stoma. CSF, corticosteroid-free; HBI, Harvey-Bradshaw Index.

Nineteen (12.6%) patients had a stoma at treatment initiation, including 12 (63.2%) patients for whom HBI data were available at baseline, 8 of which had clinically active disease at baseline. Of these, 3 (37.5%) patients were in CSFCR, 2 (25.0%) patients were nonresponders, and 3 (37.5%) patients had no data available at week 24 (Figure 3). Sensitivity analysis excluding patients with a stoma revealed no changes in mean CSFCR rates at the different time points of interest (Table S2).

#### Previous exposure to ustekinumab

Most patients (n = 124 [84.8%]) were previously exposed to ustekinumab. CSFCR at week 24 was observed in 51.7% of ustekinumab primary nonresponders (n = 15 of 29) and 56.8% of ustekinumab secondary nonresponders (n = 21 of 37). The odds of CSFCR at week was not significantly lower for ustekinumab primary nonresponders compared with secondary nonresponders (odds ratio [OR] = 1.22; 95% CI, 0.45 to 3.30; χ^2^ = 0.02; df = 1; *P* = .87). CSFCR at week 24 was observed in 61.5% (n = 8 of 13) of patients who were never exposed to ustekinumab. The odds of CSFCR were not significantly lower in ustekinumab nonresponders compared with ustekinumab-naive patients (OR, 0.68; 95% CI, 0.16 to 2.61; χ^2^ = 0.06; df = 1; *P* = .798), although the last group was rather small. Twenty (16.1%) ustekinumab-experienced patients also discontinued risankizumab treatment; 4 (20.0%) patients were primary nonresponders for both medications and 5 (25.0%) patients were secondary nonresponder for both ustekinumab and risankizumab.

#### Previous exposure to small molecules

Twenty-eight (18.5%) patients previously used at least 1 small molecule. Twenty-four (15.9%) patients were upadacitinib experienced, of which 23 (95.8%) patients showed active disease (clinical and/or biochemical) at initiation of risankizumab. At week 12, 7 (41.2%) patients were in clinical remission, while 10 either stopped using risankizumab (n = 3) or still showed signs of clinical disease activity (n = 7). At week 24, 5 (38.5%) patients were nonresponders and 7 (53.8%) patients were in CSFCR. One (7.7%) patient was in clinical remission but used systemic corticosteroids between weeks 12 and 24. Five patients reached 52 weeks of follow-up: 3 (60.0%) patients were in CSFCR and 2 (40.0%) were nonresponders.

#### Patients using additional advanced therapy at baseline

Eight (5.3%) patients concomitantly used risankizumab and another advanced therapy. All patients were using an anti-tumor necrosis factor (ie, adalimumab, golimumab, infliximab) prescribed for the treatment of rheumatologic disease (n = 4) and skin disease (n = 4). At week 24, 2 out of 4 (50%) patients were in CSFCR, while 1 discontinued risankizumab and another had clinically active disease. The other patients had either insufficient follow-up (n = 2) or missing data (n = 2) at week 24. Sensitivity analysis excluding patients using concomitant advanced therapy revealed no changes in mean CSFCR rates at the different time points of interest (Table S3).

### Predictors for CSFCR at week 24

Patients using systemic corticosteroids at treatment initiation had 67.8% lower odds of achieving CSFCR at week 24 compared with patients who did not use systemic corticosteroids at baseline (OR, 0.322; 95% CI, 0.11 to 0.87; *P* = .03). Multivariable regression model included systemic corticosteroids use at baseline, disease duration, history of bowel-related surgery, and clinical disease activity at baseline. None of these potential predictors reached statistical significance (Table S4).

### Treatment discontinuation and persistence

**Figure 4:**
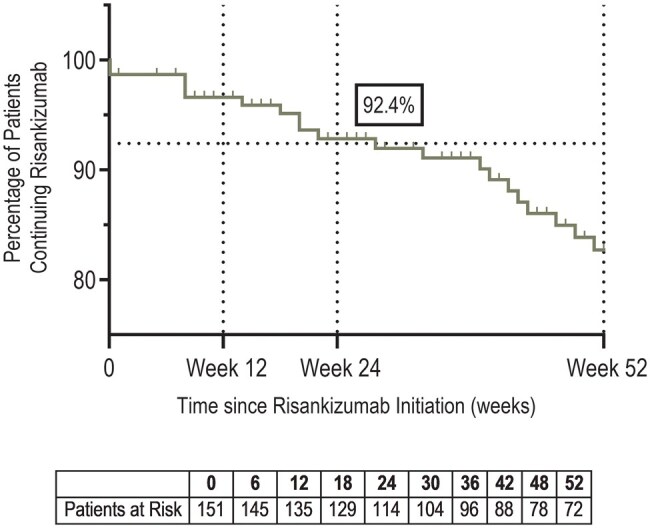
Kaplan-Meier survival analysis for evaluating risankizumab persistence up to week 52, including the number of patients at risk by time.

Cumulative risankizumab treatment survival is illustrated in Figure 4. Twenty-two patients stopped using risankizumab after a median treatment duration of 183.5 days (Q1-Q3: 90.0-295.2 days). Reasons for treatment discontinuation were primary nonresponse (n = 9 of 22 [40.9%]), serious adverse events (n = 7 of 22 [31.8%]), and secondary nonresponse (n = 6 of 22 [27.3%]) (Table 2).

**Table 3.** List of reported adverse events during risankizumab treatment.

	Number of events	Per 100 person-years
**Infections**	45	31.2
**Mild infections**	25	17.3
**Respiratory**	10	
**Flu-like symptoms**	6	
**Herpes zoster**	3	
**Cold sores**	2	
**Eye**	2	
**Gastrointestinal**	1	
**Skin**	1	
**Moderate infections**	17	11.8
**Skin**	3	
**Eye**	2	
**Herpes zoster**	2	
**Respiratory**	2	
**Urinary**	2	
**Cold sores**	1	
***Clostridioides difficile***	1	
**Fever**	1	
**Gastrointestinal**	1	
**Gynecological**	1	
**Mouth**	1	
**Severe infections**	3	2.1
**Gastrointestinal**	2	
**Respiratory**	1	
**Other adverse events**	83	57.5
**Possibly related**	51	35.3
**Skin complaints**	12	
**Gastrointestinal complaints**	8	
**Exacerbation of symptoms**	7	
**Musculoskeletal complaints**	7	
**Fatigue**	5	
**Headache**	2	
**Respiratory**	2	
**Dehydration**	1	
**Eye**	1	
**Hepatobiliary**	1	
**Leukopenia**	1	
**Nausea**	1	
**Other**	3	
**Probably related**	24	16.6
**Headache**	6	
**Skin complaints**	5	
**Fatigue**	3	
**Cold**	2	
**Respiratory**	2	
**Cold sores**	1	
**Eye**	1	
**IBD exacerbation**	1	
**Malaise**	1	
**Musculoskeletal complaints**	1	
**Leading to discontinuation**	8	5.5
**Skin complaints**	4	
**Herpes zoster**	1	
**Hyperhidrosis**	1	
**Malaise during IV administration**	1	
**Musculoskeletal complaints**	1	

Abbreviation: IBD, inflammatory bowel disease; IV, intravenous.

### Safety profile

During follow-up, 11.8 moderate and 2.1 severe infections were reported per 100 patient-years. Most reported infections were respiratory infections, herpes zoster infections, and cold sores (Table 3).

**Table 2:** Reasons for treatment discontinuation (n = 22).

Reason	
**Primary nonresponse**	9 (40.9)
**Adverse events**	7 (31.8)
**Secondary nonresponse**	6 (27.3)

In total, 35.3 possibly and 16.6 probably risankizumab-related adverse events per 100 person-years were reported during follow-up (Table 2). The most probably related adverse events were headache and skin complaints, including hair loss and rash at the site of injection.

Seven patients discontinued risankizumab due to adverse events, with 5.5 adverse events leading to discontinuation per 100 person-years. One patient had to discontinue risankizumab after having a malaise during the first intravenous. Urticaria and rash at the injection spot were considered as probably related to risankizumab in 4 patients, including a patient who also had an exacerbation of musculoskeletal complaints. However, the relation between the musculoskeletal complaints and the use of risankizumab was uncertain. One female patient suffered from hyperhidrosis; however, it was probably related to menopausal changes. Last, 1 patient experienced herpes zoster infection, which was possibly related to risankizumab.

## Discussion

We assessed the real-world effectiveness and safety of risankizumab for patients with CD up to 52 weeks of treatment in a prospective, nationwide cohort. CSFCR at week 24 was achieved in 50% of patients with clinically active disease at baseline.

These relatively high CSFCR rates were observed despite the inclusion of a highly therapy-refractory population. Over 80% of patients were ustekinumab experienced, many had failed multiple biologics and small molecules, and more than half had previously undergone bowel-related surgery or had a stoma. These findings underline the potential of risankizumab in complex, difficult-to-treat IBD, and support its use beyond the populations included in clinical trial setting. No new safety signals were identified, supporting the tolerability of risankizumab in daily clinical practice.

The efficacy of risankizumab in inducing and maintaining remission has been assessed in different phase 3 trials. Clinical remission and endoscopic improvement were observed in patients after 12 weeks of risankizumab treatment, and significantly higher clinical remission rates were observed in patients using risankizumab compared with the placebo group, independent of prior therapy failure. However, direct comparisons with our real-world data should be made with caution due to differences in inclusion criteria and baseline characteristics. Our study included patients with more advanced disease and more extensive prior treatment failure, likely reflecting broader clinical practice compared with the RCTs.

To our knowledge, this is the first prospective multicenter study to examine the effectiveness of risankizumab in patients with CD. Our findings are in line with results previously published in the first prospective, although monocenter, real world study on risankizumab in CD. We both showed that more than half of the patients with active disease at baseline were in clinical remission 6 months after risankizumab initiation.^9^ Moreover, roughly half of the patients included in both studies were in CSFCR remission at week 52. Although these percentages are comparable, it is important to mention that patients included in our study experienced more therapy-refractory disease, especially regarding prior ustekinumab failure (84.8%) compared with Zinger et al’s patients (52.2%).^9^ Other real-world studies were published, but data collection was retrospective, patient characteristics were different, and outcome definitions differed too much to properly compare the different observations.^5^^,^^6^ Our results also reflect findings from Caron et al,^12^ who described risankizumab as a good treatment option for difficult-to-treat disease.

While CSFCR is often prioritized as the principal outcome in efficacy and effectiveness studies, biochemical remission is also of considerable importance. It offers a noninvasive surrogate for mucosal healing, a therapeutic target associated with sustained clinical remission and lower rates of IBD-related complications.^13^ Zinger et al^9^ described a higher percentage of patients with FC ≤250 µg/g at each visit, including 83.3% at week 24 compared with 36.5% in our cohort. However, they reported more patients (35.4%) with FC ≤250 µg/g at baseline compared with our cohort (17.9%). Although evidence of biochemical effectiveness remains limited, these results show that risankizumab is more effective at achieving clinical than biochemical remission.

Roughly half of the patients in our cohort experienced at least 1 adverse event, including infections, while using risankizumab. This proportion is similar to the safety analysis reported in ADVANCE (A Multicenter, Randomized, Double-Blind, Placebo Controlled Induction Study of the Efficacy and Safety of Risankizumab in Subjects With Moderately to Severely Active Crohn's Disease), MOTIVATE (A Multicenter, Randomized, Double-Blind, Placebo-Controlled Induction Study to Assess the Efficacy and Safety of Risankizumab in Subjects With Moderately to Severely Active Crohn's Disease Who Failed Prior Biologic Treatment), and FORTIFY (A Multicenter, Randomized, Double-Blind, Placebo Controlled 52-Week Maintenance and an Open-Label Extension Study of the Efficacy and Safety of Risankizumab in Subjects With Crohn's Disease) trials.^14^^,^^15^ Nonetheless, the number of infections per 100 patient-years was lower in our study (31.2) than in the FORTIFY trial (51.4).^15^ Similar to the safety analysis conducted by Zinger et al,^9^ respiratory complaints were among the most reported adverse events, although more respiratory infections were reported in our cohort (13 vs 4). Interestingly, Alsoud et al^5^ mentioned that no serious or opportunistic infections were reported during follow-up; these findings contrast with our results, as several bacterial and viral infections were reported in our cohort. Our results also contrast with the low percentage (29.0%) of patients with adverse events and the unique case of infection reported by Fumery et al^6^; this difference could be caused by the retrospective study design of the French study.

The ICC Registry is designed to observe the patient journey in routine clinical practice within a multicenter, nationwide cohort, thereby maximizing generalizability of the results as much as possible to the general IBD population. However, this real-world design entails inherent limitations. Variables can be missing at certain time points due to variation in clinical care. Although it is considered the gold standard, endoscopic evaluation is not routinely used in daily practice to assess disease activity, specifically when patients are doing well. Another indicator of treatment effectiveness is therapeutic drug monitoring. While therapeutic drug monitoring is available for most biologicals in the Netherlands, it is not yet implemented for risankizumab. Additionally, data on perianal disease during follow-up were missing. Furthermore, in routine care, the different visits may take place in a wider range around the time point of interest, reflecting variability in clinical practice. Last, only a few patients already reached 52 weeks of follow-up, limiting the interpretation of the results at that time point. Restricting analyses to patients eligible for each follow-up window may have introduced selection bias, as continued follow-up may be associated with treatment response or prognosis. However, treatment failures occurring prior to the respective assessment were conservatively classified as nonresponse, thereby reducing the risk of inflating effectiveness estimates.

At the same time, the real-world design of this study provides several key strengths. We examined both clinical and biochemical outcomes. While clinical remission is important for patients’ quality of life, biochemical remission, as assessed by FC, is a marker for mucosal remission. Most importantly, our cohort was representative of patients initiating risankizumab in daily clinical care because patients were included in both academic and general centers nationwide and no restricting inclusion criteria were used, allowing us to also study patients with a stoma.

In conclusion, this multicenter prospective real-world observational cohort study demonstrates that risankizumab is an effective and safe treatment option for CD, even in the context of difficult-to-treat disease.

## Supplementary Material

izag045_Supplementary_Data

## Data Availability

The data underlying this article will be shared upon reasonable request to the corresponding author.
