# Supplementary material for: Prospective real-world experience with risankizumab in difficult-to-treat Crohn’s disease: results from the Dutch Initiative on Crohn and Colitis Registry
Source: Inflamm Bowel Dis. 2026 Apr 4;32(8):1460–70. doi: 10.1093/ibd/izag045 (PMC13414538; doi:10.1093/ibd/izag045)
Supplement: izag045_Supplementary_Data [file izag045_supplementary_data.docx]

| **Supplementary Table 1**. Predefined time windows around each visit of interest. | | |
| --- | --- | --- |
| **Timepoint** | Range around timepoint (days) | Timeline |
| Baseline (T_0_) | [- 56; 0] | T_-56_ - T_0_ |
| Week 12 (T_84_) | [- 28; 28] | T_56_ - T_112_ |
| Week 24 (T_168_) | [- 28; 56] | T_140_ - T_224_ |
| Week 52 (T_365_) | [- 28; 56] | T_337_ - T_421_ |


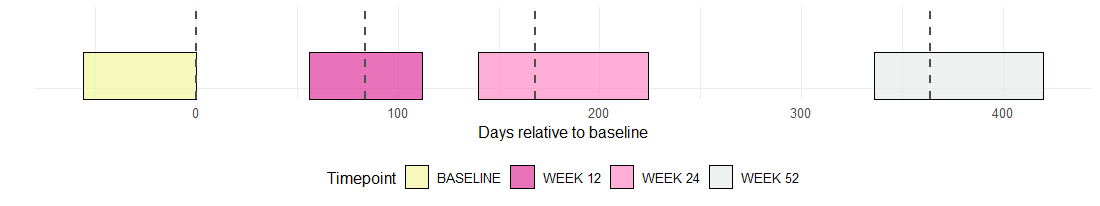


**Supplementary Figure 1**. Visual representation of the predefined time windows around each visit of interest.

**Supplementary Figure 2**. Visual representation of the patient distribution based on status and data availability at each timepoint; **a.** all patients who started risankizumab (N = 151); **b.** patients with clinically active disease at treatment initiation (N = 90); **c.** patients with difficult-to-treat disease, defined as patient who were previously exposed to advanced therapies with at least two different mechanisms of action (N = 136).

*Notes* - patients with HBI available (HBI < 5, HBI < 5 & CSF, HBI > 4) or who stopped using risankizumab (stop) were included in the denominator to compute clinical remission rates; patients with unknown HBI but using systemic corticosteroids (non-CSF) were added to the analysis of CSFCR; patients with missing data (follow-up, unknown HBI) were excluded from the analysis for the specified timepoint.

*Abbreviations* - CSF, corticosteroid-free; HBI, Harvey-Bradshaw Index

| **Supplementary Table 2**. Percentage of patients in corticosteroid-free clinical remission with and without patients with stoma at baseline. | | | | | |  |
| --- | --- | --- | --- | --- | --- | --- |
|  | Main analysis | 95% CI | Sensitivity analysis | 95% CI | Absolute difference | |
| Baseline | 38/142 (26.8) | 19.8, 34.9 | 35/127 (27.6) | 20.2, 36.3 | 0.8 | |
| Week 12 | 51/104 (49.0) | 39.2, 59.0 | 46/95 (48.4) | 38.1, 58.8 | 0.6 | |
| Week 24 | 49/91 (53.8) | 43.1, 64.2 | 44/82 (53.7) | 42.4, 64.6 | 0.1 | |
| Week 52 | 34/55 (61.8) | 47.7, 74.3 | 31/50 (62.0) | 47.2, 75.0 | 0.2 | |
| *Notes* - results presented as proportion and percentage; absolute difference between the results of the main analysis and the sensitivity analysis excluding patients with stoma at baseline reported as percentage  *Abbreviation* - 95% CI, 95% confidence interval | | | | | | |

| **Supplementary Table 3**. Percentage of patients in corticosteroid-free clinical remission with and without patients using concomitant advanced therapy at baseline. | | | | | |  |
| --- | --- | --- | --- | --- | --- | --- |
|  | Main analysis | 95% CI | Sensitivity analysis | 95% CI | Absolute difference | |
| Baseline | 38/142 (26.8) | 19.8, 34.9 | 34/134 (25.4) | 18.4, 33.7 | 1.3 | |
| Week 12 | 51/104 (49.0) | 39.2, 59.0 | 48/98 (49.0) | 38.8, 59.2 | 0 | |
| Week 24 | 49/91 (53.8) | 43.1, 64.2 | 47/87 (54.0) | 43.0, 64.6 | 0.2 | |
| Week 52 | 34/55 (61.8) | 47.7, 74.3 | 33/53 (62.3) | 47.9, 74.9 | 0.5 | |
| *Notes* - results presented as proportion and percentage; absolute difference between the results of the main analysis and the sensitivity analysis excluding patients using concomitant advanced therapy at baseline reported as percentage  *Abbreviation* - 95% CI, 95% confidence interval | | | | | | |

| **Supplementary Table 4**. Results of the univariable and multivariable regression analyses. | | | | | | |
| --- | --- | --- | --- | --- | --- | --- |
| Variable | OR | 95% CI | P value | OR | 95% CI | P value |
| Age at inclusion | 0.981 | 0.952 - 1.01 | 0.206 |  |  |  |
| Sex  Male  Female | Ref.  0.571 | 0.235 - 1.34 | 0.206 |  |  |  |
| Disease Duration | 0.967 | 0.929 - 1.0 | ***0.0765*** | 0.980 | 0.937 - 1.02 | 0.338 |
| Disease location at inclusion  L1  L2  L3 | Ref.  0.611  0.958 | 0.200 - 1.82  0.360 - 2.53 | 0.379  0.931 |  |  |  |
| Disease location at diagnosis  L1  L2  L3 | Ref.  0.500  0.636 | 0.131 - 1.81  0.260 - 1.81 | 0.294  0.406 |  |  |  |
| History of bowel resection  No  Yes | Ref.  0.496 | 0.213 - 1.13 | ***0.0979*** | 0.596 | 0.221 - 1.58 | 0.299 |
| Presence of stoma  No  Yes | Ref.  1.04 | 0.257 - 4.44 | 0.959 |  |  |  |
| Ustekinumab-experienced  No  Yes | Ref.  0.629 | 0.180 - 1.99 | 0.440 |  |  |  |
| Difficult-to-treat Diseases  No  Yes | Ref.  0.374 | 0.053 - 1.73 | 0.244 |  |  |  |
| Systemic corticosteroids  No  Yes | Ref.  0.322 | 0.110 - 0.870 | ***0.0296*** | 0.435 | 0.137 - 1.31 | 0.144 |
| HBI at baseline  > 4  ≤ 4 | Ref.  2.30 | 0.902 - 6.30 | ***0.0897*** | 2.24 | 0.848 - 6.33 | 0.113 |
| FC at baseline  > 250 µg/g  ≤ 250 µg/g | Ref.  0.636 | 0.218 - 1.81 | 0.399 |  |  |  |
| Smoking behaviour  Never smoked  Ex-smoker  Current smoker | Ref.  0.703  1.90 | 0.251 - 1.95  0.621 - 6.35 | 0.498  0.272 |  |  |  |
| *Abbreviations* - 95% CI, 95% confidence interval; FC, faecal calprotectin; HBI, Harvey-Bradshaw Index; OR, odds ratio; Ref., reference value | | | | | | |
